# Serological evidence of exposure to Rift Valley, Dengue and Chikungunya Viruses among agropastoral communities in Manyara and Morogoro regions in Tanzania: A community survey

**DOI:** 10.1371/journal.pntd.0008061

**Published:** 2020-07-20

**Authors:** Rule M. Budodo, Pius G. Horumpende, Sixbert I. Mkumbaye, Blandina T. Mmbaga, Richard S. Mwakapuja, Jaffu O. Chilongola

**Affiliations:** 1 Kilimanjaro Clinical Research Institute, Moshi, Tanzania; 2 Department of Public Health and Research, Lugalo Military College of Medical Sciences (MCMS) and General Military Hospital (GMH), Dar es Salaam, Tanzania; 3 Department of Medical Biochemistry and Molecular Biology, Kilimanjaro Christian Medical University College, Moshi, Tanzania; 4 Directorate of Research and Consultancies, Kilimanjaro Christian Medical University College, Moshi, Tanzania; 5 Tanzania Veterinary Laboratory Agency (TVLA), Kibaha, Tanzania; Institute for Disease Modeling, UNITED STATES

## Abstract

Tanzania has recently experienced outbreaks of dengue in two coastal regions of Dar es Salaam and Tanga. Chikungunya and Rift Valley Fever outbreaks have also been recorded in the past decade. Little is known on the burden of the arboviral disease causing viruses (Dengue, Rift Valley and Chikungunya) endemically in the inter-epidemic periods. We aimed at determining the prevalence of the dengue, rift valley and chikungunya among humans in two geo ecologically distinct sites. The community-based cross-sectional study was conducted in Magugu in Manyara region and Wami-Dakawa in Morogoro region in Tanzania. Venous blood was collected from participants of all age groups, serum prepared from samples and subjected to ELISA tests for RVFV IgG/IgM, DENV IgG/IgM, and CHIKV IgM/IgG. Samples that were positive for IgM ELISA tests were subjected to a quantitative RT PCR for each virus. A structured questionnaire was used to collect socio-demographic information. Data analysis was performed by using SPSSv22. A total of 191 individuals from both sites participated in the study. Only one individual was CHIKV seropositive in Magugu, but none was seropositive or positive for either RVFV or DENV. Of the 122 individuals from Wami-Dakawa site, 16.39% (n = 20) had recent exposure to RVFV while 9.83% (n = 12) were seropositive for CHIKV. All samples were negative by RVFV and CHIKV qPCR. Neither infection nor exposure to DENV was observed in participants from both sites. Being more than 5 in a household, having no formal education and having recently travelled to an urban area were risk factors associated with RVFV and CHIKV seropositivity. We report a considerable exposure to RVFV and CHIKV among Wami-Dakawa residents during the dry season and an absence of exposure of the viruses among humans in Magugu site. In both sites, neither DENV exposure nor infection was detected.

## Author summary

Dengue (DENV), Chikungunya (CHIKV), Rift Valley (RVFV) viruses and other viruses constitute an important group of etiologies of fever in Tanzania. In the past decade, Tanzania has experienced DENV and RVFV outbreaks at different times. While RVFV outbreaks have occurred cyclically in approximately ten-year periods in Tanzania, DENV outbreaks have been more frequent since 2010. CHIKV infection is an important but largely unrecognized illness in Tanzania. In this study, we aimed to generate baseline data on the exposure and infection status of DENV, RVFV and CHIKV by detecting antibodies to the viruses and detecting the viruses in human subjects in two geo-ecologically distinct sites. Neither infection nor exposure to the viruses was in observed in Magugu site in Manyara region, northern Tanzania. There was a significant exposure to RVFV (16.39%) and CHIKV (9.83%) in Wami-Dakawa but not to DENV in either site. None of the viruses was detected by PCR in any of the sites. Potential risks for exposure to CHIKV and RVFV were larger numbers of household members, having no formal education and having recently travelled to an urban destination. Since arboviral outbreaks are usually unpredictable, it is crucial to undertake active surveillance for RVFV, DENV, CHIKV and other viral agents in Tanzania.

## Introduction

RVFV, DENV and CHIKV are endemic in Sub-Saharan Africa and cause sporadic and sometimes large epidemics in humans. The ecological drivers of the pattern and frequency of virus infections (and subsequent epidemics) in the different host species are largely unknown[1]. Many authors identified non-human primates, and birds as hosts/reservoirs for arboviruses[2–4]. Although Arboviruses are genomically variant, they share a common transmission mode through vectors, pathobiological mechanisms and cause overlapping clinical presentations[5]. DENV and CHIKV are principally transmitted by *Aedes Aegypti* mosquitoes, but occasionally they can be transmitted by *Ae albopictus* and *Ae*. *Polynesiensis* while RVFV is mainly transmitted by floodwater Aedes sp including *Ae*. *Mcintoshi* and *Ae. Ochracius [6–8]*. DENV and CHIKV are an increasing global public health concern due to their rapid geographical spread and increasing disease burden.

The transmission of these viruses is complicated by human population migration/resettlement, internal displacement due to environmental and civil unrest in many African countries and the triad of the modern world: urbanization, globalization, and international mobility[1]. CHIKV causes large epidemics with serious economic and social impact and its clinical presentations are similar to several flavivirus infections[9–12]. Dengue virus has caused major epidemics in Southeast Asia and Southern American countries in past centuries [8]. In the Americas, dengue was effectively controlled in the mid-1900s by effective control of *A*. *aegypti*, the principal urban vector of both viruses. However, there has been a re-emergence of dengue worldwide[13]. Previous studies demonstrated that some degree of DENV genetic variability is necessary for the persistence during periods of endemicity and epidemic outbreaks[14]. For decades, arboviral diseases were considered to be of minor concern with regard to global mortality and disability. Consequent to this, low priority has been given to arboviral research and related public health infrastructure. In the past decade, however, an unprecedented emergence of epidemic arboviral diseases (notably dengue, chikungunya, and Rift valley fever) has been recorded in Tanzania. Earlier studies conducted in north-eastern Tanzania showed that the prevalence of dengue and Chikungunya were 7.9% and 12.9%, respectively [12, 15]. The Rift valley fever outbreaks have occurred in 39.1% of districts in Tanzania through the period of 1930–2007 [6]. The most recent outbreak in 2007 was associated with human and livestock morbidity as well as significant mortalities and abortions in livestock. However, there are no reports describing the occurrence of arbovirus infections, such as DENV, CHKV, and RVFV[12]. It is important to understand the prevalence of arboviruses in different weather conditions in Tanzania in order to ascertain their presence in different hosts at different weather conditions annually.

Arboviruses are primarily maintained by horizontal transmission (HT) between arthropod vectors and vertebrate hosts in nature. Occasionally, they are transmitted vertically in the vector population from an infected female to the offspring, which is a proposed maintenance mechanism during adverse conditions [10]. For RVFV, it is known that factors such as dense vegetation, suitable temperature conditions, and the presence of ruminants make it favorable for mosquitoes to breed, replicate the virus, and pass it on to animals and humans. The recent epidemics caused by these arboviruses have been associated with many factors including urbanization/population growth and international travel and trade, allowing for spread of vectors, and spreading of arboviruses into new niches, followed by amplification through human-vector-human cycle. Climate change is predicted to further impact on the distribution of vector-borne diseases, such as Rift Valley fever and dengue, which are highly sensitive to climatic conditions [16]. The maintenance mechanisms during inter epidemic periods (IEPS) become interesting as to where the viruses hide during the “silent” periods. This study was therefore, carried out to establish exposure and infection status of RVFV, DENV and CHIKV in two geo-ecologically distinct sites in North Eastern and Central Eastern Tanzania during the dry season to generate baseline data that will form the basis of a future ‘across-season’ transmission study.

## Materials and methods

### Ethics statement

Ethical clearance was sought from Kilimanjaro Christian Medical University College Research and Ethics Review Committee (CRERC) and clearance certificate number 2419 was obtained. Permission to conduct the study was obtained from both Manyara and Morogoro Regional Administrative Secretaries. The Babati Rural and Mvomero District Executive Officers and District Medical Officers were consulted for permission.

### Study design, study sites and sample size estimation

#### Study design and sample size

A cross-sectional serological survey was conducted in two purposively selected wards Magugu in Babati rural in Manyara region and Wami-Dakawa in Mvomero district in Morogoro region June-August 2019. Households were conveniently included in the study. Participants were interviewed using a structured questionnaire. The sampling process involved a two-stage purposive selection of districts and wards based on findings of past studies that reported the status of RVF outbreaks in Tanzania. The selection of wards was not based on statistical considerations, but on logistic and resource availability. Selection of participants was based on availability (haphazard) sampling, and thus the study involved 191 participants in both study sites, 69 participants from the Magugu site whereas 122 participants were from Wami-Dakawa site.

#### Study sites

**Fig 1:**
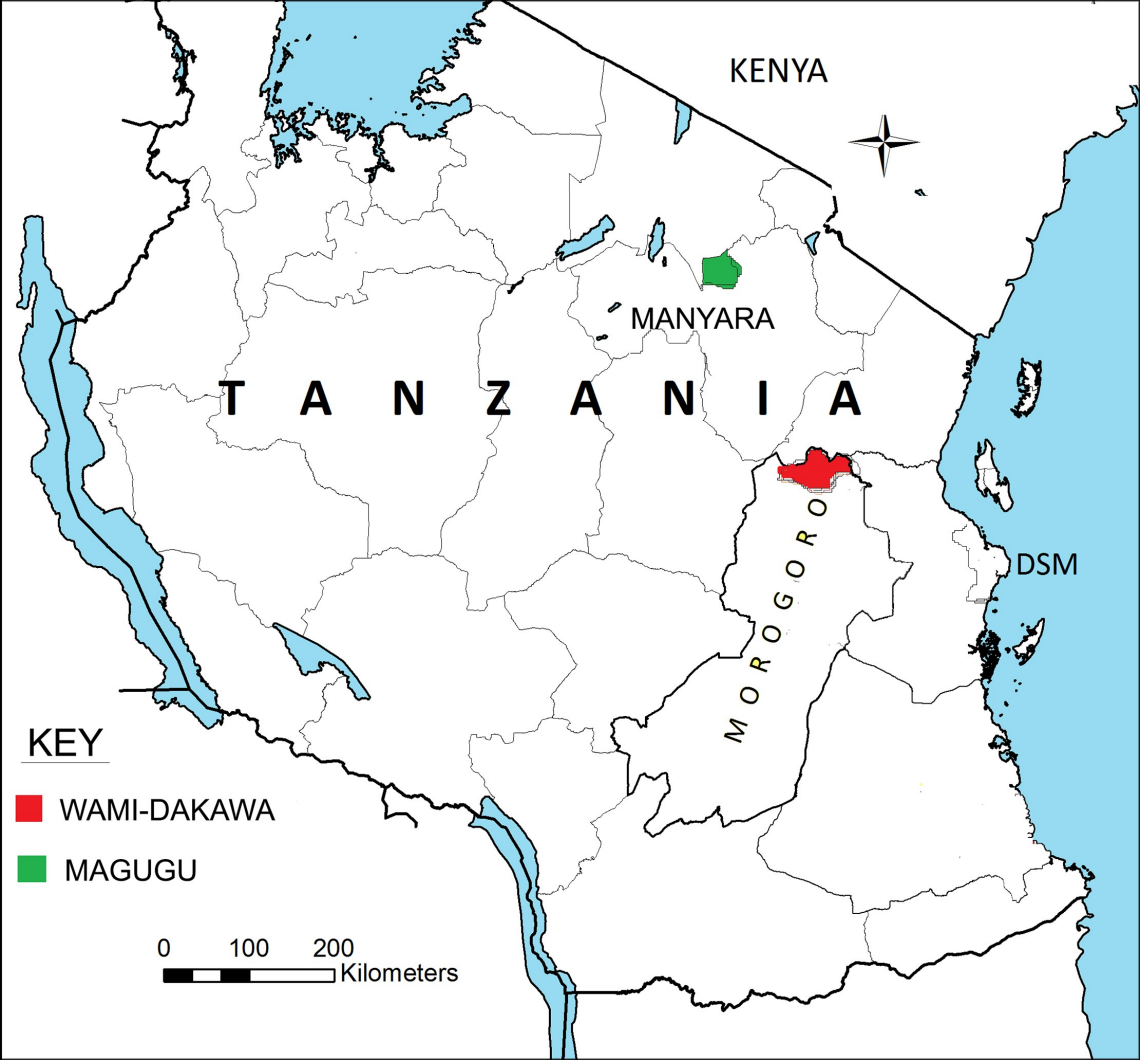
Map of Tanzania showing the study sites (Magugu and Wami-Dakawa). Map developed by authors using ArcGIS software, version 10.8

The current study involved two purposively selected ecologically distinct areas where significant human-mosquito-livestock interaction exist as the key criterion that makes it very feasible for studies on transmission of RVFV, DENV and CHIKV. The selection was intended to maximize the chances of detecting the viruses among residents of the sites. Magugu site is located in Manyara Region in Northern Tanzania and Dakawa site in Morogoro region located in Eastern-Central Tanzania. (Fig 1)

Magugu ward (35°46′00″E;4°01′00″S) is located in Babati Rural District, in northern Tanzania along the eastern rift valley, at 1013m above sea level. The main economic activities include crop production and livestock keeping. There is one government owned health center which serves all the seven villages. Average annual rainfall is about 650mm. There are two rainy seasons, short rainy season from October to December and long rainy season from mid-March to May, followed by a cool and dry season from June to mid-August, and a hot dry period from mid-August to October. Wami-Dakawa (37°28'13"E; 6°35'2.8"S) is a ward in Mvomero district of Morogoro region. The area has semi humid climate with an average annual rainfall of 800 mm. The short rains start in November and end in January followed by heavy rainfall between March and May and a dry season from June to October. Similar to Magugu site, the main economic activities in Wami-Dakawa is crop production and livestock keeping.

#### Participants

This study included adult residents (≥18 years), males and females of the two sites who were either peasants or livestock keepers and willing to participate in the interviews. Salaried and other individuals whose primary occupation was not either agriculture or livestock keeping were excluded from participating in the study.

#### Blood samples collection, processing and storage of specimens

From each subject, 3ml of blood was collected from median cubital vein following venipuncture. The blood sample was equally aliquoted into a plain vacutainer (1.5ml) and 1.5 ml was placed into EDTA tube and an equal volume of Trizol added as per manufacturer’s instructions (Zymo Research, Irvine, CA, U.S.A.). The EDTA tube mixture was gently mixed for 5 minutes and immediately both samples were kept at 4°C in cool boxes before they were shipped to the laboratory. A standardized questionnaire was used to collect clinical and socio-demographic information from participants.

### Laboratory procedures

#### DENV IgG/IgM and CHIKV IgM ELISAs

Briefly, serum from plain tubes was obtained by centrifugation of samples at 2,000 rpm x g for 10 minutes in a refrigerated centrifuge. Serum samples were stored at −20°C awaiting serological analyses. For seropositivity of CHIKV, anti-CHIKV IgM were analyzed using an Indirect ELISA kit (SD, Gyeonggi-do, Korea and IBL international, Hamburg, Germany, respectively). Detection of DENV IgG and IgM antibodies were done using a direct enzyme linked immunosorbent assay (ELISA) kit (SD Inc, Gyeonggi-do, Korea) as described by [17]. All assays were performed according to manufacturers’ instructions. Optical density (OD) reading was done at 450 nm and the units of antibody concentration and cut-off values were calculated as described by the manufacturers. Briefly, for the Anti-DENV IgM/IgG and IgM anti-CHIKVELISAs, the diagnostic cut-off value was calculated as the average OD of negative controls + 0.300. For the IgM CHIKV ELISA the threshold for positivity was based on the OD cut-off value of the control + 10%.

#### RVFV competitive ELISA (cELISA)

All samples were tested for antibodies against RVFV using a competitive ELISA (ID Screen Rift Valley Fever Competition Multi Species, ID-vet, Grables, France), which detects both IgG and IgM antibodies directed against the RVFV nucleoprotein (NP). Validation tests for the test has shown to have a sensitivity of between 91 and 100% and a high specificity of 100%. The competitive ELISA was performed according to the instructions of the manufacturer and as described previously [18]. In order to control the validity of each plate, the mean value of the two negative controls (OD_NC_) was computed whereby a plate was considered valid if the OD_NC_ was>0.7. For a valid plate, the mean value of the two positive controls divided by OD_NC_ had to be <0.3. For each sample, the competition percentage was calculated by dividing OD_sample_/OD_NC_) × 100. If the value was equal or less than 0.4, the sample was considered positive while a value greater than 0.5 was considered negative.

#### Ribonucleic acid extraction and RT-PCR procedures

For DENV and CHIKV, Blood samples kept in EDTA tubes were centrifuging at 1,000 rpm x g for 10 minutes in a refrigerated centrifuge to obtain buffy coat. Ribonucleic acid (RNA) was extracted from buffy coat samples using the Boom method [18]. cDNA were synthesized using Superscript VILO cDNA synthesis kit (Invitrogen, life technologies, USA) according to manufacturer’s instructions and PCR done as previously described[17]. For RVFV detection, RNA was extracted from sera using a QIAamp viral RNA mini kit (QIAGEN, Germany) as per manufacturer’s instructions. RVFV RNA was detected using TaqMan probe-based one-step RT-PCR targeting the RVFV Gn gene as described by Gudo and colleagues [15].

#### Nature of data and data analysis

All data were collected strictly following good clinical practice (GCP) guidelines. Participants were identified using anonymous IDs and initials as the only way of their identification. The main tool for data collection was the questionnaire. Data that were collected were participant demographics, characteristics of households, characteristics of livestock herds, treatment history, travel, co-morbidities, virologic and serological parameters. Data were analyzed using SPSS v.24(IBM Corp., Armonk, NY, USA). Descriptive data are reported as frequencies while categorical data are reported as tabulation of proportions. Logistic regression analyses were used to examine associations between seropositivity to RVFV, DENV and CHIKV and candidate predictor variables. Bivariate models were constructed for candidate demographic variables and herd characteristics evaluated. Only variables with a coefficient p-value ≤0.2 in the bivariate model were considered in the multivariate modelling as previously suggested [19]. Only adjusted odds ratios (aORs) from the multivariate model were reported.

## Results

### Descriptive statistics

A total of 191 samples were collected, 122 from Wami-Dakawa and 69 from Magugu. Since only one CHIKV case was detected by serology among the 69 samples collected from Magugu site, presented results include only samples collected from Wami-Dakawa site. Of the122 individuals who participated in the study from Wami-Dakawa, 58(47.5%) were involved in pastoralism as their main occupation while 64 (52.5%) were peasant farmers. About two thirds of the participants (65.6%) live in households with less than 5 family members. About three-fifth (60.7%) of the interviewed participants, reported not to have travelled outside Wami-Dakawa during the previous three months, with 33/48 (68.8%) of those who had travelled having travelled to an urban destination (Table 1). A total of 60 (49.2%) participants had herd sizes made of less or equal 30 goats. Goat browsing was mainly done in communal grasslands by 64.8% of the participants. Browsing was done about 70 km from Mikumi national park/game reserves, with minimal interaction between livestock and wild animals (Table 2). Out of the 122 people tested for RVFV, 20 (16.39%) were positive for IgM/IgG antibodies to RVFV, this indicate that there is active circulation of RVFV in the area. Twelve of the participants (9.83%); tested positive for anti-CHIKV IgM (Table 3). Only one participant was seropositive for CHIKV IgM from Magugu site. Through interviews, the patient who was seropositive for CHIKV IgM in Magugu reported to have arrived in Magugu 3 weeks prior to the sampling from Tanga region. All samples that were positive for anti-RVFV and anti-CHIKV IgM antibody tested negative for qPCR.

**Table 1:** Demographic characteristics of participants.

Family Characteristics	Number	Percent
Sex		
Female	49	40.2
Male	73	59.8
Age in years (mean = 41.4, SD = 13.6, min = 20, max = 97)		
≤30	25	20.5
31–50	73	59.8
>50	24	19.7
Occupation		
Agriculture	64	52.5
Pastoralism	58	47.5
Number of people in household under one roof		
<5	80	65.6
≥5	42	34.4
Travel out of domicile in past three months		
No	74	60.7
Yes	48	39.3
If yes, mention where		
Rural	15	31.25
Urban	33	68.75
Highest education received		
Primary School or Higher	43	35.3
No formal education	79	64.8
Marital status		
Single	14	11.5
Living together	108	88.5

**Table 2:** Characteristics and grazing practices of participants.

Herd Characteristics	Number	Percent
Herd size		
≤30	60	49.2
31–50	12	9.8
>50	50	41.0
Characteristic of grazing area		
Forest	41	33.6
Plaingrasslands	79	64.8
Savannah	2	1.6
Do you graze very close to game reserve		
No	121	99.2
Yes	1	0.8
Do you camp your animals during dry season		
No	104	85.3
Yes	18	14.8
Do you meet wildlife where you take your herd for water drinking?		
No	103	84.4
Yes	19	15.6
Distance of grazing in kilometers		
≤10	92	75.4
Nov-20	4	3.3
≥31	26	21.3
Distance of water source in kilometers		
≤10	94	77.1
Nov-20	2	1.6
≥31	26	21.3
Distance from wildlife in kilometers		
≤10	61	50.0
Nov-20	1	0.8
21–30	22	18.0
≥31	38	31.2

**Table 3:** Enzyme linked immunosorbent assay results for IgM/IgG antibodies to Rift Valley Fever Virus and Chikungunya viruses.

Total tested	Rift Valley Fever IgG/IgM seropositivity				Chikungunya IgM/IgG seropositivity				
	+ve	%	95% C.I.		Total tested	+ve	%	95% C.I.	
			Low	Up				Low	Up
122	20	16.39	9.8	23.6	122	12	9.83	4.9	15.7

### Risk factors for Rift Valley Fever Virus IgG/IgM seropositivity

Compared to participants aged 30 years or less, participants aged between 31 and 50 were least likely to be seropositive for RVFV IgG/IgM [OR 0.64; (95%CI: 1.8–2.33), p<0.05]. Participants aged 51 years and above were more likely to be RFVF IgM/IgG seropositive compared to participants who were less than 30 years old, although this difference was not statistically significant [OR 2.15, (95%CI: 2.43–5.1), p<0.05]. Households with more than 5 people sleeping in the same house were 2.15 times more likely to be RVFV IgG/IgM seropositive compared to households with less than 5 people [OR 1.35, (95%CI: 0.33–5.56), p<0.678]. Similarly, participants without formal education had higher odds of being RVFV IgG/IgM seropositive compared to participants with formal education [OR 1.9, (95%CI: 2.63–4.56), p<0.05]. (Table 4).

**Table 4:** Risk factors for RVFV IgG/IgM Seropositivity.

Predictors of IgG/IgM	aOR	P-value	95% C.I.	
			Lower	Upper
Age in years				
≤30	1			
31–50	0.64	0.050	1.8	2.33
>50	1.35	0.678	0.33	5.56
Occupation				
Agriculture	1			
Pastoralism	0.77	0.608	0.29	2.08
Place travelled				
Rural	1			
Urban	1.68	0.553	0.30	9.34
no of people				
<5	1			
≥5	2.15	0.038	2.43	5.10
Education				
Primary school or higher	1			
No formal education	1.90	0.029	2.63	4.56

### Risk factors for Chikungunya IgM seropositivity

Results in Table 5 show that, participants who had recently travelled to an urban place were 3 times more likely to be seropositive for CHIKV IgM antibody as compared to participants who had travelled to a rural destination [OR 3.0, (95%CI: 3.3–27.6), p<0.05]. Households with 5 or more members living in the same house were more likely to be seropositive for CHIKV IgM antibody as compared to households with less than 5 people [OR 3.5, (95%CI: 1.6–4.84), p<0.05]. Likewise, participants with lack of formal education had higher odds of being seropositive for CHIKV compared to those with formal education [OR 2.85, (95%CI: 2.4–5.0), p<0.05].

**Table 5:** Risk factors for Chikungunya IgM seropositivity.

Predictors of Chikungunya	aOR	P-value	95% C.I.	
			Lower	Upper
Age in years				
≤30	1			
31–50	1.25	0.788	0.25	6.37
>50	0.83	0.861	0.11	6.46
Occupation				
Agriculture	1			
Pastoralism	1.12	0.855	0.34	3.68
Place travelled				
Rural	1			
Urban	3.00	0.033	3.3	27.60
no of people				
<5	1			
≥5	3.5	0.033	1.6	4.84
Education				
Primary school or higher	1			
No formal education	2.85	0.041	2.4	5.00

## Discussion

The aim of this study was to investigate the prevalence of antibodies against DENV, CHIKV and RVFV and to detect the presence of the viruses in humans. The study also pursued to identify potential predictors of infection by the viruses and seropositivity to the viruses during the inter-epidemic period (IEP). Results indicate that only one participant was seropositive for CHIKV among the 69 participants from Magugu. However, the results indicated that significant number of people were seropositive for RVFV and CHIKV in Wami-Dakawa whereas none of the participants was positive for DENV by either serology or PCR from either site.

Tanzania has experienced outbreaks due to DENV and RVFV for five years consecutively, from 2010–2015[20–22]. Previous studies have reported the presence of antibodies to RVFV in different parts of Tanzania during the IEP. A study conducted by Swai and Schoolman reported the prevalence of antibodies to RVFV in Tanga region [23]. A cyclical recurrence of RVF in Tanzania has been documented whereby the disease has been recurring in approximately ten-year cycles with three major epidemics reported in 1977, 1997/98 and 2006/2007[9]. The areas affected by the epidemics have been reported to vary, whereas RVF epidemics of 1977 and 1997/98 were mainly confined to northern regions of Tanzania. The 2006/2007 outbreak assumed a larger scale, involving 10 out of the 21 regions of Tanzania with human cases reported in Arusha, Dar es Salaam, Dodoma, Iringa, Manyara, Mwanza, Morogoro, Pwani, Singida and Tanga Regions[24]. Studies have not been able to detect the viruses in its hosts (mosquitoes, ruminants and human hosts) during IEPs.

Despite the failure to detect RVFV by PCR, we report a seroprevalence of 16.39% for RVFV antibodies among residents of Wami-Dakawa site, which indicates significant RVFV transmission in the Wami-Dakawa area even if the epidemiological cycles and modalities of this circulation remain unknown. Classically, the RVFV cycle involves domestic animals (livestock) as viral amplifying hosts before transmission to humans, as has previously been shown in many African countries[25]. The ecology of the Wami-Dakawa area and life sustaining economic activities (agropastoralism) of the residents of Wami-Dakawa offers suitable conditions for active transmission and maintenance of RVFV in the area due to the intimate contact between humans, livestock and vector mosquitoes. Despite the positive serological results for RVFV, this study did not diagnose any RVFV among participants by PCR. This raises the question of how the viruses are maintained across seasons and their hosts during inter epidemic periods. A clear definition of the transmission dynamics of RVFV would constitute a prime target for the control of RVF and other vector borne viral infections.

We detected none of the studied viruses by PCR, and observed an absence of seropositive individuals to any of the viruses in the Magugu site except a single, an immigrant CHIKV seropositive case. Interesting as this observation may be, the relative “sterility” of the Magugu site to mosquito borne infections warrants an epidemiological explanation owing to the otherwise conducive environment for arboviral transmission including the presence of suitable climate and breeding sites for mosquitoes. A number of reasons could explain this observation in our study, including the absence of mosquito species composition in the area and the limited sample size. Previous studies on mosquito borne infections in Magugu had reported a similar state of sterility for mosquito borne infections for the past decade[26–30].

Recent epidemiological data on CHIKV and DENV outbreaks in Tanzania has largely been reported in Dar-es Salaam, the commercial and largest city in Tanzania [20] and Tanga region [17], both cities located along the Indian Ocean coast. There have been no reported DENV and CHIKV epidemics since the reported 2014–2015 outbreaks. Recently in 2019, a DENV outbreak occurred involving largely Dar es Salaam and its neighboring regions of Pwani, Morogoro and Tanga regions.(https://www.iamat.org/country/tanzania/risk/dengue). Our study could not detect any seropositive or actively infected individuals in Wami-Dakawa. This finding was unexpected due the closeness of Wami-Dakawa and Morogoro urban that involves a rich day to day human interactions. The absence of data on presence or abundancy of Aedes mosquitoes in the current study, provides an avenue for future studies on vector abundance in relation to transmission of mosquito borne viruses in the area.

In this study, there was a higher proportion of individuals who were seropositive to RVFV (16.39%) than individuals seropositive to CHIKV (9.83%), although the difference was not statistically significant. While RVFV has non-human domestic animals as one of its hosts, CHIKV and DENV do not infect livestock, making the former easier to maintain among high risk communities, such as livestock herders, during IEPs. Sampling for this study was conducted during the dry season. It is worthwhile to recommend that this variation is followed up in tandem with entomological screening, across different seasons of the year to get an insight of how infection and seroconversion may vary as a means of RVFV, DENV and CHIKV maintenance in the absence of outbreaks.

Certain factors were associated with higher rates of RVFV infection as determined by IgM/IgG competitive ELISA, including household size of more than five persons and having no formal education. Age between 30 and 50 years was associated with a lowest risk of being RVFV seropositivity. Contrary to RVF, CHIKV seropositivity was associated with recent travel to an urban destination, household size of more than five persons and having no formal education. RVFV is a mosquito borne, viral zoonotic infection whose transmission involves both vector mosquitoes and intimate contact with tissues of affected animals. The higher likelihood of RVFV seropositivity among adults compared to young individuals has been previously reported[8, 31]. The involvement of older individuals in animal grazing, butchering, birth assistance to their animals and disposal of aborted fetuses have been mentioned as important factors for being seropositive for RVF[31].

CHIKV has been reported to have a long history of emergence into urban transmission cycles from its original, enzootic, sylvatic foci in Sub-Saharan Africa, most recently spreading to the Americas beginning in 2013. The factors shared in common between CHIKV and that predispose them to emerge into urban epidemic cycles are thought to be their moderate infectivity for *A*. *aegypti*, whose anthropophilic behavior, ecology and tendency to feed on multiple human hosts during a gonotrophic cycle[7] is ideal for transmission of a human arboviral pathogen and their ability to use primates as enzootic amplification hosts. Both DENV and CHIKV are commonly transmitted by mosquito vectors that belong to the genus Aedes (Ae.), particularly *Ae*. *aegypti* and *Ae*. *Albopictus*.

Factors that are associated with DENV and CHIK transmission have previously been mentioned to occur in urban areas [17, 20]. There are many factors that are thought to have contributed to the emergence of DENV and CHIKV epidemics, which include urbanization, globalization and insufficient mosquito control strategies [6].The study sites in this study were mainly rural area, justifying the possibility of absence of conducive conditions for transmission of the viruses as evidenced by the absence of any active DENV and CHIKV case.

Although our study is limited by the small sample size due to convenience and logistical factors, our findings provide valuable information regarding the rate of exposure to RVFV, DENV and CHIKV in the studied areas. While the absence of entomological data in this report may be a limitation, it provides an avenue for future studies to relate inter-host transmission dynamics and vector mosquito abundance.

### Conclusion and recommendation

We report antibodies to RVFV to be the most prevalent followed by antibodies to CHIKV in Wami-Dakawa, in Mvomero district of Morogoro region during dry season. This study did not detect any individual in Wami-Dakawa who was seropositive to DENV. Magugu site was found to be free from both infection and exposure to RVFV, CHIKV and DENV. Larger numbers of household members in a house, having no formal education and having recently travelled to an urban destination were risk factors being seropositive CHIKV whereas being more than 5 individuals in a household, having no formal education were the only risk factors for RVFV seropositivity. Since arbovirus outbreaks occur sporadically and usually unpredictable in nature, it is crucial to undertake active surveillance measures for RVFV, DENV, CHIKV and other viral agents in endemic countries. In addition, since our study has revealed a considerable seroprevalence to RVFV in the Wami-Dakawa study area, it is unarguably important that vaccination and surveillance systems for RVFV are strengthened to reduce RVFV transmission between animals and humans, which poses as a public health concern. This strategy is urgent in the present time characterized with drastic climatic changes.
